# Pattern Recognition for Selective Odor Detection with Gas Sensor Arrays

**DOI:** 10.3390/s121216262

**Published:** 2012-11-23

**Authors:** Eungyeong Kim, Seok Lee, Jae Hun Kim, Chulki Kim, Young Tae Byun, Hyung Seok Kim, Taikjin Lee

**Affiliations:** 1 Environment Sensor System Research Center, Korea Institute of Science and Technology (KIST), Hwarangno 14-gil 5, Seongbuk-Gu, Seoul 136-791, Korea; E-Mails: eungyeong@kist.re.kr (E.G.K.); slee@kist.re.kr (S.L.); jaekim@kist.re.kr (J.H.K.); chulki.kim@kist.re.kr (C.L.K.); byt427@kist.re.kr (Y.T.B.); 2 Department of Information and Communication Engineering, Sejong University, 98 Gunja-Dong, Gwangjin-Gu, Seoul 143-747, Korea; E-Mail: hyungkim@sejong.ac.kr

**Keywords:** gas sensor array, odor monitoring, pattern recognition, artificial neural networks (ANN), genetic algorithm (GA), neural-genetic classification algorithm (NGCA)

## Abstract

This paper presents a new pattern recognition approach for enhancing the selectivity of gas sensor arrays for clustering intelligent odor detection. The aim of this approach was to accurately classify an odor using pattern recognition in order to enhance the selectivity of gas sensor arrays. This was achieved using an odor monitoring system with a newly developed neural-genetic classification algorithm (NGCA). The system shows the enhancement in the sensitivity of the detected gas. Experiments showed that the proposed NGCA delivered better performance than the previous genetic algorithm (GA) and artificial neural networks (ANN) methods. We also used PCA for data visualization. Our proposed system can enhance the reproducibility, reliability, and selectivity of odor sensor output, so it is expected to be applicable to diverse environmental problems including air pollution, and monitor the air quality of clean-air required buildings such as a kindergartens and hospitals.

## 1. Introduction

Gas sensors are used widely in many different fields such as the prevention of natural disasters, environmental monitoring of automobile industry outputs, and other pollution-related industries [1–4]. Many types of gas sensors are used for gas detection. In particular, a semiconductor gas sensor is used to detect specific gases based on the variation in resistance or thermal conductivity due to absorption or desorption on the surface of an oxide semiconductor [5,6]. Semiconductor gas sensors are rapid, and they have simple structures with low manufacturing costs. However, they have several weaknesses in terms of accuracy and selectivity. Thus, achieving stability and reliability while maintaining the advantages mentioned above are very important requirements [7]. Improvements in stability, high resolution, repeatability, and economic feasibility need to be implemented to fulfill these requirements [8–15].

In [16], Fau *et al.* described a new and promising method for implementing micromachined silicon gas sensors, which also had very stable baseline resistance and gas sensitivity over time. In [17], the features of periodically measured data derived from thermal variation in a system based on a sensor were extracted using the fast Fourier transform (FFT), which were used to estimate a single gas concentration. In [18], the discrete wavelet transform (DWT) method was used to classify a mixture of gases. In [19], improvements in sensor selectivity and accuracy were achieved using a combination of a sensor array with a neural network. The signals from multisensors were evaluated using principal components analysis (PCA) [20,21] and artificial neural networks (ANNs), while the selection of possible features using a genetic algorithm (GA) facilitated classification in [22]. Environmental applications such as pollution monitoring and air quality control [23–25] have steadily attracted considerable attention because of the growing need for environmental protection in recent years. In addition, many recent studies have reported successful applications based on ANNs [26–29] and GAs [30,31], which were used in this study. Other pattern recognition approaches have been suggested, such as a kernel method, the drift compensation method, and a merged method based on maximum margin classification and regression [7,32,33]. Pattern recognition has also been performed based on the transient features of a metal oxide sensor’s response to a constant concentration of analytes [12]. This feature is available much earlier than the standard steady-state feature, but it delivers comparable results in quantification and identification problems.

In this study, we build a gas monitoring system to process sensed signals and to identify noxious gases. The odor monitoring system was designed to detect the patterns in the features of certain gases using a pattern recognition method that was developed for accurate data classification while online data learning was conducted for accurate prediction. A neural-genetic classification algorithm (NGCA) is introduced for pattern recognition; this algorithm integrates GA and ANN and delivers improved reliability and selectivity during pattern extraction for noxious gases. We performed data visualization via PCA [34] and verified our proposed system by comparing the results obtained using ANN, GA, and NGCA. The experimental results showed that our proposed system had a faster response speed and higher selectivity with the gas sensor.

## 2. Materials and Methods

### 2.1. Odor Monitoring Systems and Data Preprocessing

**Figure 1.**
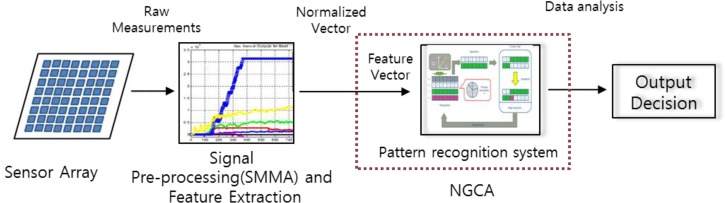
Pattern recognition system structure.

Odor monitoring systems that use gas sensors require continuous observation because of the presence, movement, or disappearance of odors. In this study, we aimed to construct an environment management system for the rapid detection of odors and for finding fundamental solutions by analyzing the data and tracking the source. In general, subtle fabrication processes and detailed signal processing techniques can guarantee high selectivity, but with a high cost [35]. To deliver high selectivity with a low cost, we developed the overall procedure for our proposed system shown in Figure 1.

**Figure 2.**
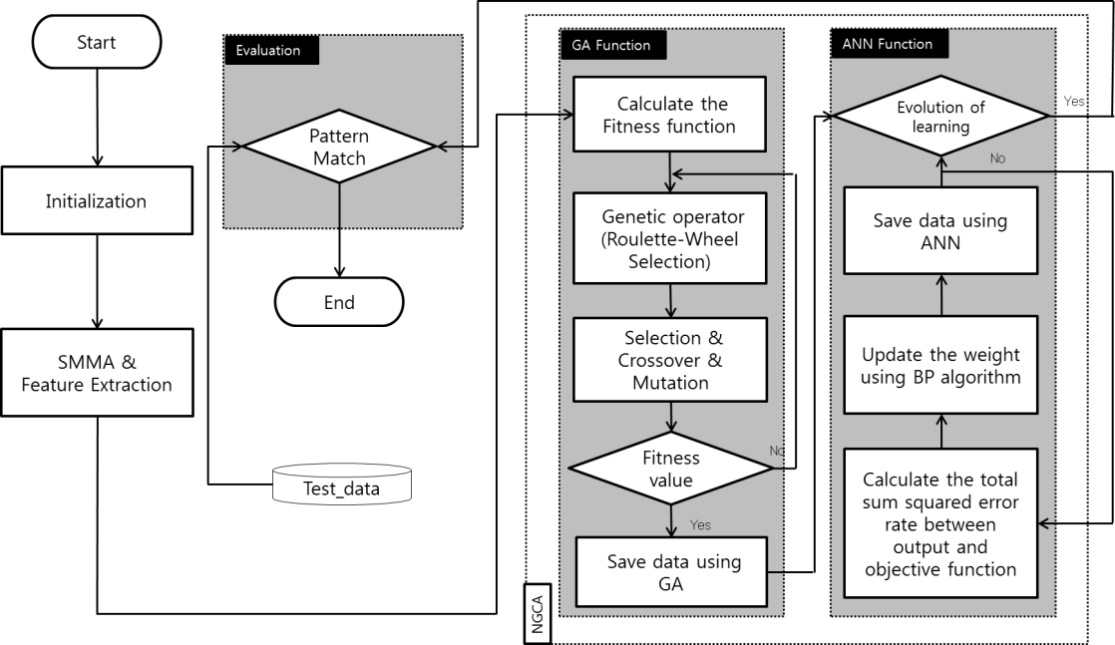
Flow of the pattern recognition algorithm used for NGCA.

Our proposed pattern recognition procedure consists of data signal acquisition from the sensor array, signal preprocessing with smoothed moving average (SMMA), and NGCA (Figure 1). Signal preprocessing and SMMA facilitate data smoothing, noise elimination, and filtering. There is no perfect single learning algorithm, so we developed NGCA by integrating GA and ANN. Figure 2 shows the detailed procedure used by our system. Our system consists of SMMA and feature extraction, NGCA (GA function and an ANN function), and an evaluation procedure. The SMMA and feature extraction processes perform following two functions: (1) the preprocessing of raw data and (2) extracting the specific pattern variation in the data. SMMA identifies and removes noise from the measured gas sensor data and returns the mean value while eliminating the oldest data and adding the newest data over time, so the filtering result varies with the time interval. As mentioned earlier, the gas sensitivity problem is generally not solved using a single identifier, so our proposed NGCA uses multiple specialized identifiers to improve the classification performance. The GA function, *i.e.*, the first process in NGCA, extracts the data with high fitness after noise filtering. The ANN function was implemented using the back propagation algorithm. The final model is constructed based on datasets collected during repeated experiments under the same conditions, e.g., sensors, materials, and room temperature. Finally, the evaluation procedure compares changes in the rate of the sensing data and the new value (final sensing) returned by NGCA in the databases. The NGCA outputs are saved in a database to allow comparisons with the true values.

### 2.2. Feature Extraction and Classification

The selection of a characteristic pattern vector is an important but difficult task when analyzing the odor patterns obtained from gas sensors. In addition to the choice of pattern recognition algorithm selected, the individual features that form a pattern have a decisive effect on the recognition rate. The analysis of the data obtained from gas sensors is a challenging task because these sensors commonly interact with many other gases that have similar molecular structures. Therefore, we developed a pattern recognition system based on NGCA, which can classify noisy gas data. The steps in the pattern recognition algorithm are as follows:

Generate the initial population for GA
Execute the GA algorithm
Execute the ANN algorithm
Execute the evaluation procedure

#### 2.2.1. Generation of the Initial Population for GA

The initial population used by the GA is created using the following procedure with the original values from eight sensors. The tangent gradient (change in rate of the sensors) is obtained after signal preprocessing, and it is used by the GA to create new data with a high fitness value. Eight different gases (O_3_, LPG/LNG, NO_x_, alcohol, smoke, VOC, CO, and NH_3_) are used as the input features to express one chromosome in the GA. All of the sensor values are normalized as real numbers between 0 and 1 by dividing them with the saturation value (*i.e.*, the maximum value).

#### 2.2.2. GA Algorithm

The fitness, *f(x)*, is used to decide that the preprocessed data in the GA is well noise-reduced. Each data point is assigned a particular fitness value based on GA chromosome selection criteria. The fitness can be expressed as follows:

(1) $$\begin{array}{c} \text{f}(\text{x})=\text{x}+\text{K}*\left|\text{sin}\left(32\text{x}\right)\right|,\;0\leq \text{x}<\pi \left(1\right)\\ \text{K}=\text{window}-\text{size} \end{array}$$

where *x* is a candidate solution and *K* is a constant that represents the window size determined during the signal preprocessing stage. Equation (1) returns a high score for a large *x* and a low score for a small x. If the fitness value is less than a fitness threshold (=0.5), the GA repeats the genetic operations, including selection, crossover, and mutation, to produce a high fitness.

We adopt an elitist preserving strategy, in which the strongest individual in a population is passed to the next generation without any changes. Crossovers are used to generate new data where the values of each chosen point selected are exchanged. There are several different types of crossover method, e.g., one point crossover, multipoint crossover, and uniform crossover. The crossover points used during crossover operations are selected randomly.

#### 2.2.3. ANN Algorithm

The gradient generated by the GA is passed to the ANN. After the ANN is built, its outputs are compared with the user-defined thresholds. If they satisfy the threshold, the data features are extracted using the evaluation procedure and are compared with the data stored in the database during the evaluation. Otherwise, the ANN is rebuilt according to the following procedures. The connection weights are reinitialized, and the learning pattern pairs are selected. The learning rate and error limits are set by changing the connection weights as appropriate. The parameters are set as listed in Table 2. After the parameters have been set, the output and error values of the hidden and output layers are calculated. An error is calculated by comparing the target value with the output value. In our experiments, we used Equation (2) with a revised target value that was suitable for changing the connection weight to generate a target value, as follows:

(2) $$d=\frac{\left(\sum_{i=1}^{m}\frac{\left|Y_{i}-P_{i}\right|}{Y_{\max}}\right)}{m}$$

where *m* is the total data number, *Y_max_* is the output of the max value, *Y_i_* is the *i-th* output, and *p_i_* represents the *i-th* predicted value.

The target value is generated using the difference between the output (*y*) and the predicted value (*p*). The obtained target value is compared with the output value to calculate the error. The error between the output and hidden layers is calculated and learned. The connection weight generated by the learning evaluator is saved as new data. This step is repeated until the threshold and the learning evaluation results are satisfied.

After the learning evaluation, the gradients (change in rate of the sensors) of the inputs and the test data are estimated as follows (calculate the gradient whenever t is a multiple of *K*):

- The gradient of the test data

(3) $$\begin{array}{c} \begin{array}{l} T_{td}\left(t\right)=\frac{\left(S_{td}\left(t\right)-S_{td}\left(0\right)\right)}{t}\left(t\leq K\right)\\ \text{and} \end{array}\\ T_{td}\left(t\right)=\frac{\left(S_{td}\left(t\right)-S_{td}\left(t-K\right)\right)}{K}\left(t>K\right) \end{array}$$

where *T_td_*(*t*) = *gradient of test_data*, *S_td_(t)* is the SMMA for the interval *t*, and *K* is the window size.

- The gradient of the input

(4) $$\begin{array}{c} \begin{array}{l} T_{id}\left(t\right)=\frac{\left(S_{id}\left(t\right)-S_{id}\left(0\right)\right)}{t}\left(t\leq K\right)\\ \text{and} \end{array}\\ T_{id}\left(t\right)=\frac{\left(S_{id}\left(t\right)-S_{id}\left(t-K\right)\right)}{K}\left(t>K\right) \end{array}$$

where *T_tid_*(*t*) = *gradient of Input_data*, *S_id_(t)* is the SMMA for the interval *t*, and *K* is the window size.

#### 2.2.4. Evaluation Procedure

The evaluation procedure was designed to determine the performance of the NGCA. The evaluation procedure compares the sensor values (unseen) for eight gases saved in the database with the data created using the NGCA by computing the success rate using a similarity value. The following evaluation procedure was used to determine the identity of the sensed gas:

***Step 1***: Data from the input sensors is used for initialization by matching the database data.
***Step 2***: Evaluate the data from the input sensors and the database data.
  ***Step 2_1***: Calculate the gradient (rate of change of the sensing data) measurement form epoch to epoch.
  ***Step 2_2:*** Compare the gradient and the final sensing data in the database.
  ***Step 2_3***: Calculate the success rate.
***Step 3***: If at least six among eight gas sensors have the same pattern and more than 80% in all epoch, a successful identification is performed.

## 3. Results and Discussion

### 3.1. Gas Sensor Set Up

We used an array module containing eight different types of gas sensors to measure the odor. If the target gas was mixed with other gases, the gas sensor array containing different sensors was more efficient when measuring a specific gas than a single element. All of the experiments were performed indoors to minimize variation in the environmental conditions, e.g., temperature, humidity, and the natural drift of the semiconductors. We successfully improved the recognition rate using NGCA. At the same time, it provided more reliable quantitative data on the gas. In the experiment, we used the following gases: O_3_, LPG/NG, NO_x_, alcohol, smoke, VOC, CO, and NH_3_. The output signals from the sensors entered the analog multiplexer. Table 1 lists the specifications of each sensor. The output signal from each sensor could be controlled using variable resistors in the gas sensor array module.

### 3.2. System Set Up

**Figure 3.**
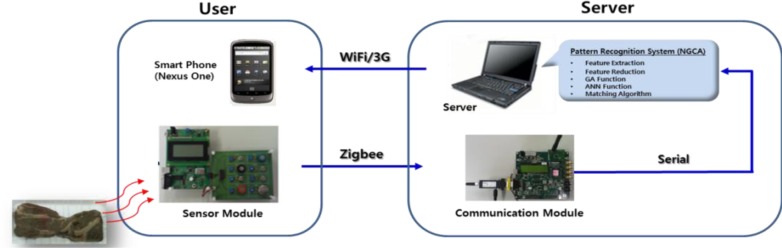
Detection system based on a gas sensor array and a smartphone.

The sensing system consisted of a smartphone, a sensor array module, and a server computer. The sensor array module contained a ZigBee wireless communication module and eight different commercial sensors. The signals from the eight different sensors were ADC-processed by the MUX at 1 Hz. The digitized sensing values were transmitted to the server computer via ZigBee communication. This system used ZigBee communication because it uses very small power and a relatively small data size is needed for processing. The sensor computer used the pattern recognition module of the NGCA, as shown in Figure 3. The server computer performed the pattern recognition process, and the results were sent to users’ smartphone via Wi-fi or using the WCDMA communication protocol so that they could recognize the identity of the sensed material. The smartphone used was a Nexus One unit (Google Company) with an Android OS while a laptop PC was used as the server.

### 3.3. NGCA Parameters

Features were extracted from the filtered data using the NGCA. Of the 50 events measured, 30 events were used as the training dataset whereas the remainder were used as the test dataset. The data were used to train the ANN, which was trained using the back propagation algorithm. We used Matlab for the computer simulation. Table 2 lists the parameters used in the experiment.

### 3.4. Experimental Results

**Figure 4.**
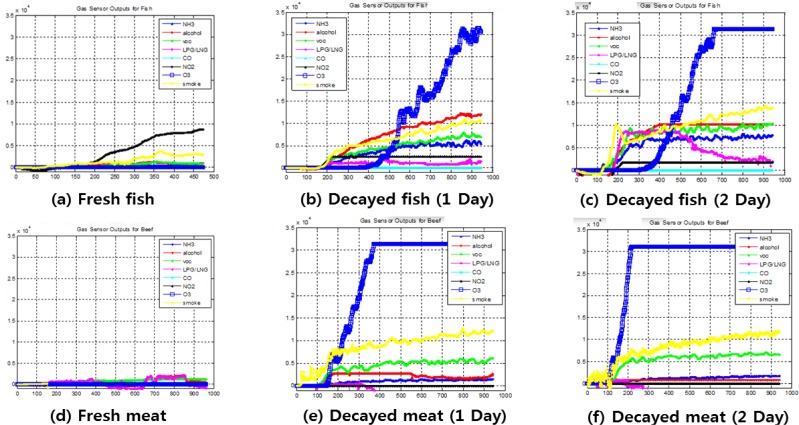
Comparison of the output of beef and fish (mackerel). (**a**) Fresh fish. (**b**) Decayed fish (1 day). (**c**) Decayed fish (2 days). (**d**) Fresh meat. (**e**) Decayed meat (1 day). (**f**) Decayed meat (2 days).

Food such as meat, fish, and shellfish decay rapidly and may cause food poisoning. This experiment evaluated the relationship between the odor parameters and the decay time. Samples of fish and meat were allowed to decay at room temperature. Odor data were collected from the decaying samples and stored in the database each day while the food was kept at room temperature for four days. We combined the absolute value of the odor strength and the differences in the measurements during pattern recognition. This approach aimed to take advantage of the different measurements from the gas sensors because of their different sensitivities to various substances. Figure 4 shows the gas sensor outputs for beef and mackerel, where there were similar patterns for the two substances. The *x*-axis and the *y*-axis represent the time (s) and voltage, respectively.

Alcohol, VOC, smoke, and O_3_ sensors were used to assess the decomposition of meat. In Figure 4(a–c), different responses of the sensors and the different mackerel decomposition rates are shown where the selectivity of the sensors was too low for the mixture gas. Similar results to the fish experiment were achieved with meat, which are shown in Figure 4(d–f).

**Figure 5.**
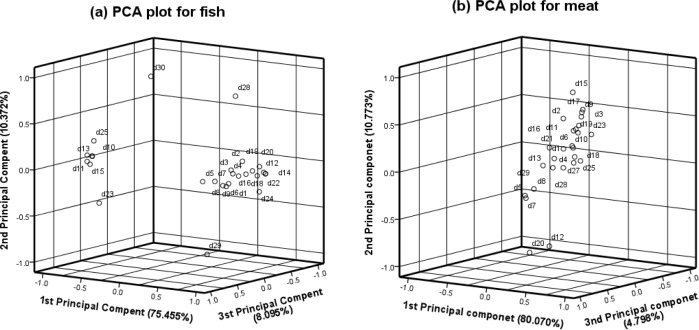
PCA plots of the 30 training datasets showing the three principal components derived from the PCA: (**a**) fish and (**b**) meat.

**Figure 6.**
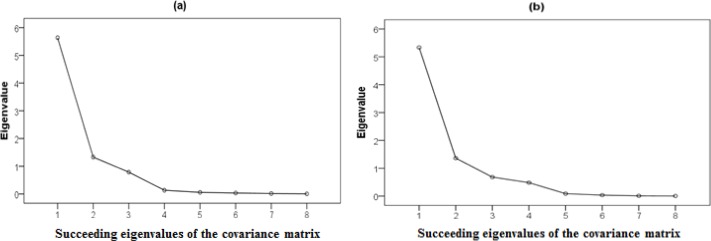
Average eigenvalues for the 30-sample training dataset: (**a**) fish and (**b**) meat.

In the experiment, we obtained 1,000 datasets, each of which contained an array of sensor values during a particular time frame (1–1,000 s). We repeated this experiment 50 times using the same fish and meat as in the previous experiment. A single dataset contained the series of values from the eight sensors at a specific time. Next, we artificially created a single dataset by averaging the 1,000 observations in the dataset for each experiment. Figure 5 shows the distribution of 30 training datasets in terms of three principle components derived from the PCA. The gas data are the averaged extracted patterns from the decay of fish and meat, which were measured for four days. Figure 5 shows distinct data distributions of fish and meat. Figure 6 shows the average eigenvalues for each feature in the 30 training datasets. The eigenvalue of the first feature was significantly higher than that of the second feature.

To identify the substances detected, the data were compared with the data in the database. The measured outputs from the same sensors at room temperature with the same parameters were analyzed using various algorithms including existing GA and ANN methods, as well as the ANN in NGCA and the GA in NGCA (our proposed NGCA methods). To ensure a valid comparison of algorithms, the GA and ANN methods included in the MATLAB toolbox were utilized. We also tested the ANN in NGCA and the GA in NGCA, *i.e.*, our proposed NGCA methods, which were modified from the GA and ANN in MATLAB. Table 3 summarizes the performance of the algorithms, as the success rate with an unknown test substance.

The success rate was 82% with ANN, 91% with GA, 92% with the ANN in NGCA, 72% with the GA in NGCA, and 95% with NGCA. This showed that the method proposed in this study delivered the best matching performance. We confirmed that the mixed ANN and GA method gave a higher success rate than ANN or GA alone. Thus, our proposed algorithm delivered accurate results based on pattern extraction with a high success rate.

## 4. Conclusions

In this study, we have developed an odor monitoring system that delivered improved pattern recognition using our NGCA approach and a semiconductor gas sensor array to detect harmful gases and environmental hazards. Our aim was to mitigate the selectivity and reliability problems that occur when measuring multiple gases simultaneously. We achieved this aim by using a pattern recognition algorithm that combined ANN and GA, which we refer to as NGCA.

Our novel NGCA pattern recognition module can extract patterns with greater reliability. The proposed system is an intelligent odor monitoring system for monitoring various odors that occur in hazardous situations. We tested the performance of our system by monitoring the odors of meat and fish samples, which were maintained at room temperature for four days. We found that the data patterns of fish and meat varied every on a daily basis. To validate the proposed method, we compared the success rate using ANN, GA, and NGCA. Our proposed method, NGCA, delivered a higher success rate than GA and ANN. We suggest that our proposed pattern perception approach may be applied to various environmental problems such as air pollution.

## Figures and Tables

**Table 1.** Specifications of the gas sensors.

**Sensor Property**		**Detecting Materials**	**Heater Voltage**	**Circuit Voltage**	**Road resistance**	**Power**
**Sensor**	**Sensor Model**					
O3	MICS-2610	O_3_	5.0 V	Max. 24 V	Variable	833 mW
LPG/LNG	GSLS-11	Smoke, Alcohol, Butyl acid	5.0 V	5 V	100 kΩ	680 mV
NOx	GSNT-11	Smoke, Alcohol, Hydrogen, CO	5.0 V	420 mW	Variable	220 mW
Alcohol	MQ-3	Benzene, CH4, Hexane, LPG, CO	5.0 V	5 V ± 0.1	Variable	less than 750 mW
Somoke	GSAP-61	HC, VOC, Methane, Butane Alcohol, TMA, Toluene, Acetaldehyde	5.0 V	Less than 12.0 V	Variable	Less than 760 mW
VOC	GSBT-11	Alcohol, Butyl acid, Hydrogen, Smoke, HC, VOC	5.0 V	5 V	2 KΩ	360 mW
CO	GSET-11	Alcohol, Smoke, Hydorgen Butane, HC	5.0 V	5 V	400 kΩ	450 mW
NH3	TGS-826	Iso-butane, Hydrogen, Ethanol	5.0 V	Max. 24 V	Variable	833 mW

**Table 2.** Parameters used in the experiment.

**[a] ANN Parameter**		**[b] GA Parameter**	
	
**Parameter**	**Value**	**Parameter**	**Value**
Input	8	Population size	1,500
Hidden layer	flexible	Generation	200
Output	1	Crossover Probability	18% 0.18)
Learning Rate	0.01	Mutation Probability	1% (0.01)
Momentum	0.2	Length of Chromosome	flexible
Learning goal	0.0002		

**Table 3.** Results using ANN, GA, ANN in NGCA, GA in NGCA, and NGCA.

**Comparison**	**ANN**	**GA**	**ANN of NGCA**	**GA of NGCA**	**NGCA**
Result (%)	82%	91%	92%	72%	95%
